# The Indoor Mycobiomes of Daycare Centers Are Affected by Occupancy and Climate

**DOI:** 10.1128/aem.02113-21

**Published:** 2022-03-22

**Authors:** Eva Lena F. Estensmo, Synnøve Smebye Botnen, Sundy Maurice, Pedro M. Martin-Sanchez, Luis Morgado, Ingeborg Bjorvand Engh, Klaus Høiland, Inger Skrede, Håvard Kauserud

**Affiliations:** a Section for Genetics and Evolutionary Biology (Evogene), Department of Biosciences, University of Oslo, Oslo, Norway; b Oslo Metropolitan University, Oslo, Norway; c Naturalis Biodiversity Center, Leiden, Netherlands; d Mycoteam AS, Oslo, Norway; Centers for Disease Control and Prevention

**Keywords:** built environment, daycare center, dust, fungi, indoor air, mycobiome

## Abstract

Many children spend considerable time in daycare centers and may be influenced by the indoor microorganisms there, including fungi. In this study, we investigate the indoor mycobiomes of 125 daycare centers distributed along strong environmental gradients throughout Norway. Dust samples were collected from doorframes outside and inside buildings using a community science sampling approach. Fungal communities in the dust samples were analyzed using DNA metabarcoding of the internal transcribed spacer 2 (ITS2) region. We observed a marked difference between the outdoor and indoor mycobiomes. The indoor mycobiomes included considerably more yeasts and molds than the outdoor samples, with *Saccharomyces*, *Mucor*, *Malassezia*, and *Penicillium* being among the most dominant fungal genera. Changes in the indoor fungal richness and composition correlated with numerous variables related to both outdoor and indoor conditions; there was a clear geographic structure in the indoor mycobiome composition that mirrored the outdoor climate, ranging from humid areas in western Norway to drier and colder areas in eastern Norway. Moreover, the number of children in the daycare centers, as well as various building features, influenced the indoor mycobiome composition. We conclude that the indoor mycobiomes in Norwegian daycare centers are structured by multiple factors and are dominated by yeasts and molds. This study exemplifies how community science sampling enables DNA-based analyses of a high number of samples covering wide geographic areas.

**IMPORTANCE** With an alarming increase in chronic diseases like childhood asthma and allergies, there is an increased focus on the exposure of young children to indoor biological and chemical air pollutants. Our study of 125 daycares throughout Norway demonstrates that the indoor mycobiome not only reflects cooccurring outdoor fungi but also includes a high abundance of yeast and mold fungi with an affinity for indoor environments. A multitude of factors influence the indoor mycobiomes in daycares, including the building type, inhabitants, as well as the outdoor environment. Many of the detected yeasts and molds are likely associated with the human body, where some have been coupled with allergies and respiratory problems. Our results call for further studies investigating the potential impact of the identified daycare-associated mycobiomes on children’s health.

## INTRODUCTION

Over a short historical time period, humans have moved from a mostly outdoor lifestyle to spending a large part of their life in indoor environments. Although the diversity of other co-occurring organisms is considerably lower in indoor environments, humans are not alone. If moisture and organic materials are available indoors, fungi can grow and disperse spores. Some of the most prevalent fungi that are able to grow and sporulate in houses include various ascomycete molds such as *Cladosporium*, *Penicillium*, and Aspergillus (1, 2). Fungal growth can lead to poor indoor air quality, and some of these fungi are associated with allergic reactions (3–5) and respiratory disease symptoms (6, 7), which may have long-term impacts on human health. Furthermore, certain combinations of indoor fungi and bacteria in moisture-damaged buildings may also cause negative health effects, even at low concentrations (8).

In many countries, children spend considerable time in daycare centers, where they are exposed to indoor microorganisms, including fungi. Since young children often bring in organic materials such as soil and litter from nature, daycare centers may accumulate extra organic substrates promoting fungal growth, compared to other indoor environments. In line with this, it was previously shown that the concentration of fungi in daycare centers is higher than that in homes (9). In several studies, the outdoor environment has been reported to be the main source of indoor fungi (10–13) due to the influx of spores through windows, entrances, and the ventilation system. Hence, the vegetation and climate that structure the outdoor fungi will indirectly also structure the indoor mycobiome (11). In correspondence with this, in a recent DNA metabarcoding study performed in 271 private homes across Norway, we showed that outdoor climate was one of the main drivers of the indoor dust mycobiome (13). A similar observation was made by Barberán et al. from dust samples collected on the outside surfaces of homes across the United States (11).

In addition to the outdoor environment, the inhabitants and their diverse activities, the presence of pets and plants, as well as various building features may contribute to and structure the indoor mycobiome (14, 15). Many yeasts, such as *Malassezia* and *Candida*, are associated with the human body and may therefore be prevalent indoors (16–19). Which fungi are associated with the human body may, to some extent, be age dependent. For instance, the basidiomycete yeast *Malassezia* seems particularly prevalent on adults (20), while children tend to have a more diverse skin-associated mycobiome, including genera like Aspergillus, *Epicoccum*, *Cladosporium*, Cryptococcus, and *Phoma*, in addition to *Malassezia* (18).

The indoor mycobiome can be analyzed in different ways, including the isolation and cultivation of fungi, microscopy, and different molecular analyses. DNA metabarcoding, based on high-throughput sequencing of PCR-amplified markers, is established as an effective approach to survey fungal communities (21). In buildings, DNA metabarcoding of dust samples, integrating spores and hyphal remains that have accumulated over time, has proven to be an effective means for exploring the indoor mycobiome (10, 12, 13, 22, 23). However, it might be difficult to get access and obtain samples from a representative number of buildings. By providing detailed instructions, dust samples can alternatively be collected by the inhabitants themselves, from which DNA can be extracted and analyzed further (13, 24). This type of community-based research, where networks of nonprofessionals help to collect data as part of a research project, is regarded as community science (also often termed citizen science) (25–27). Sampling through community science is a powerful approach, where sample equipment can be sent out by post, returning hundreds or even thousands of samples covering large geographic areas.

**FIG 1:**
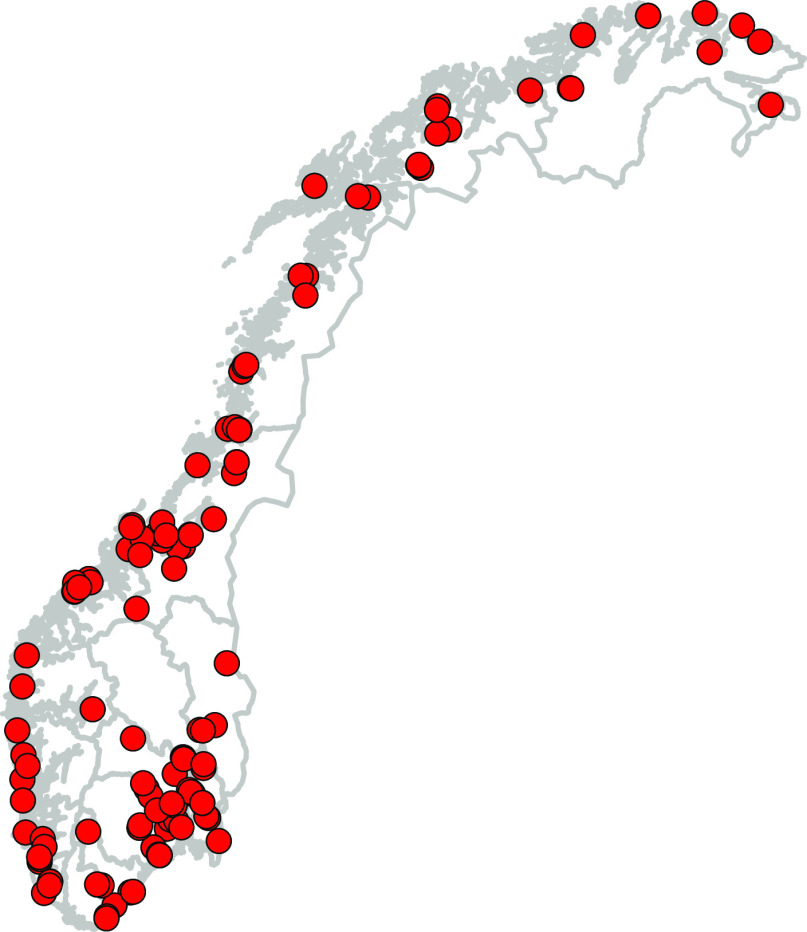
Map of Norway showing the geographic locations of the 125 daycare centers included in the study. The samples were collected by community scientists, including samples from both indoor and outdoor environments.

The impact that long-term exposure to indoor fungi can have on human health highlights the need to better characterize the indoor mycobiome from an early age. In this study, we aim to analyze the indoor mycobiomes associated with daycare centers. We ask (i) which outdoor and indoor factors drive the daycare mycobiome and (ii) which fungal groups dominate in the daycare centers, compared to outdoor samples. To address these research questions, we chose a community science approach where daycare personnel collected dust samples according to our instructions. We obtained 572 samples from doorframes inside (bathroom and main room) and outside (main entrance) from 125 daycare centers throughout Norway (Fig. 1). Norway spans extensive gradients in climate and other environmental factors, enabling us to evaluate the influence of the outdoor environment on the indoor mycobiome, in addition to building features and inhabitant characteristics. The obtained dust samples were analyzed by DNA metabarcoding of the ribosomal DNA (rDNA) internal transcribed spacer 2 (ITS2) region.

## RESULTS

### Factors influencing the indoor mycobiome.

**FIG 2:**
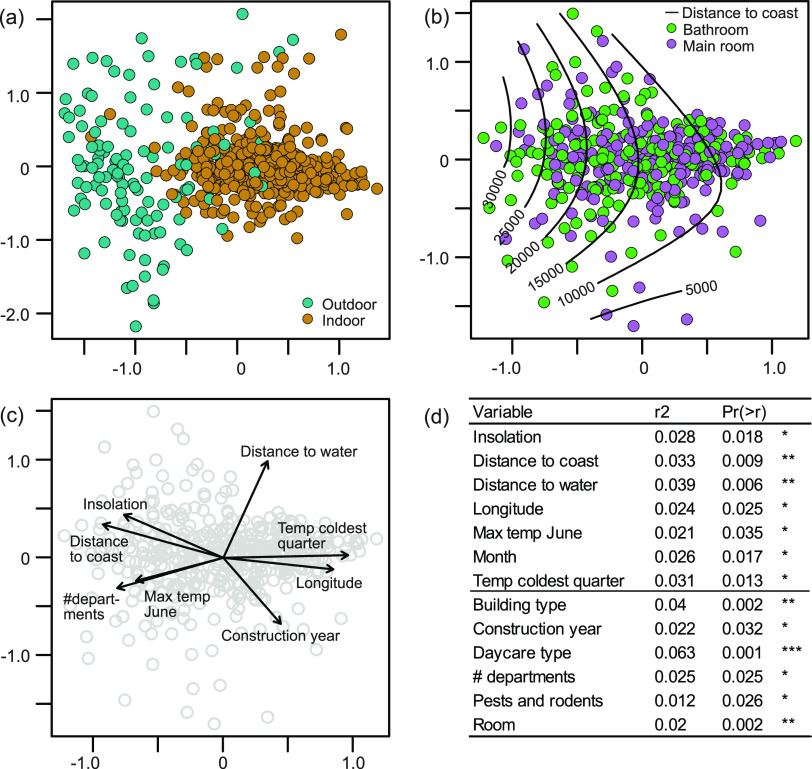
Fungal community composition in daycare centers. (a to c) Ordination plots displaying compositional variation in the dust mycobiome, where each point indicates one dust sample. (a) NMDS plot displaying both outdoor and indoor samples. (b) NMDS plot of only indoor samples from bathrooms and main rooms. The isolines represent the distance to the coast. (c) Indoor samples with vectors representing numeric variables with significant associations with the compositional variation in the indoor mycobiome (*P* < 0.05). Categorical variables are not displayed. (d) Goodness-of-fit statistics (*r*^2^) for variables that significantly (*P* < 0.05) account for the variation in the composition of the indoor mycobiome. Variables related to regional climate are listed in the top part of the table, while variables related to the specific daycare centers are listed in the bottom part. The asterisks indicates the level of significance.

Our final rarified data set from the 125 daycare centers included 748,836 sequences, with 1,342 sequences in each of the 558 samples from indoor and outdoor environments. A total of 5,946 fungal operational taxonomic units (OTUs) were detected in the data set. In a multivariate (nonmetric multidimensional scaling [NMDS]) analysis, we observed a relatively clear separation between the outdoor and indoor dust mycobiomes (Fig. 2a). However, the two types of indoor samples, main room versus bathroom, largely overlapped in fungal community compositions (Fig. 2b). Through a questionnaire to the community scientists (personnel of the daycare centers), we obtained information about different building and occupancy variables (Table 1). In addition, information about the local climate and vegetation was extracted based on the geographic coordinates of the daycare centers (28). Considered individually, many of these variables correlated significantly with the compositional variation in the indoor mycobiome (Fig. 2c), including variables related to the daycare centers, such as daycare type, construction year, number of departments, pests, and building type. Climatic variables such as temperature and total insolation were also significantly correlated with the indoor mycobiome composition, as were spatial variables that likely mirror additional regional environmental variability (Fig. 2c and d). Many of the inferred variables were associated with the major climate gradient stretching from humid, oceanic areas in western Norway to inland, continental areas in eastern Norway (Fig. 2c and d). Evaluation of the relative contribution of variables together in a canonical correspondence analysis (CCA) (Table 2) revealed that longitude (mirroring the regional climate gradient), the presence of pests/rodents, the year of construction of the daycare center, and the number of children were the main drivers of the fungal community composition, with very small interaction effects (<0.01%). However, these factors accounted altogether for only 7% of the variation in mycobiome composition (Table 2). The indoor fungal richness, calculated on a sample basis, was significantly higher in the bathroom than in the main room, and there was a significant positive correlation between indoor fungal richness and the maximum outside temperature during May at the sampling location as well as the proximity to the coast (see the mixed-effect model presented in Table 3).

**TABLE 1:** Climatic and building metadata selected by a correlation test (|*r*| > 0.6)^*a*^

Variable	Category type
Area	Categorical: urban/rural
Mean temp of the coldest quarter	Numeric
Max June temp	Numeric
Max May temp	Numeric
Proximity to all water bodies	Numeric
Proximity to coast	Numeric
Longitude	Numeric
Sampling month	Categorical: March–May
Age of children in the sampled department	Numeric
Building material	Categorical: wood/brick/concrete
Building type	Categorical: detached house/semidetached house/block/collection of buildings
Construction year	Numeric
Moisture problems	Categorical: yes/no
No. of children	Numeric
No. of departments	Numeric
Pests/rodents	Categorical: no/mouse/rat/grey silverfish/other
Ventilation type	Categorical: natural/mechanical/balanced
Water damage	Categorical: yes/no

a The upper part of the table includes the six first climatic variables extracted from a database (28) using georeferences of the daycare centers. The variables about the occupants and building features provided by volunteers in each daycare center are listed in the lower part of the table.

**TABLE 2:** Variables with explanatory power in the canonical correspondence analysis^*a*^

Variable	Variation explained
Longitude	0.0159
Pests/rodents	0.0187
Construction year	0.0181
No. of children	0.0156
Interaction effects	0.0001
Unexplained variation	0.9316

a Note that these variables may reflect a combination of variables or represent other variables not necessarily inferred here.

**TABLE 3:** Richness analyses using a mixed-effect model with the number of OTUs per sample as a response and daycare as a random effect^*a*^

Variable	Estimate	SE	*t* value	*P* value
Room (bathroom = baseline)	−3.0773	1.310563	−2.348083	0.0195
Proximity to coast	0.000095	0.000043	2.193266	0.0291
Max May temp	1.671905	0.55933	2.98912	0.003

a For the variable room type, bathroom is in the baseline of the model; the estimate for room represents the difference from the bathroom to the main room.

### Taxonomic composition of daycare mycobiomes.

**FIG 3:**
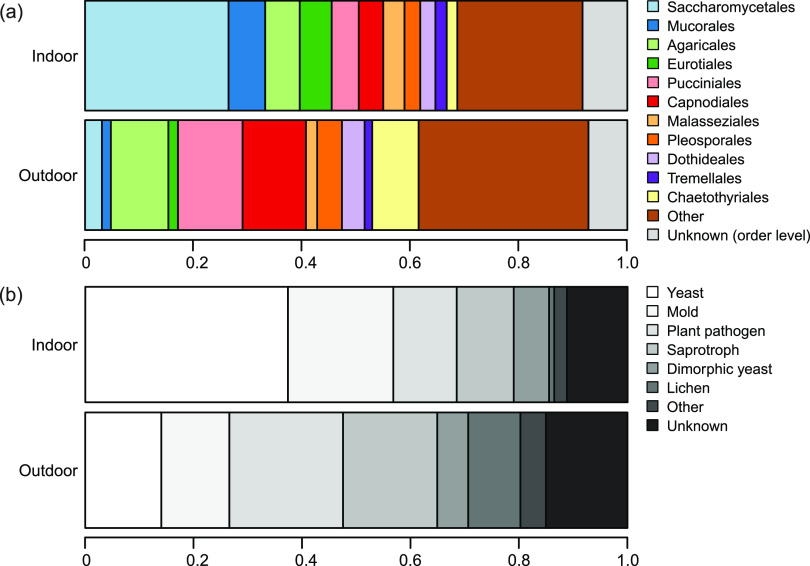
Taxonomic distribution of fungal OTUs in outdoor and indoor dust samples from the daycare centers reflecting sequence numbers. (a) Relative abundances of the main fungal orders based on the rarified OTU table. (b) Relative abundances of the same OTUs annotated as different growth forms/nutritional modes. The category saprotroph represents litter and wood decay fungi.

In the indoor mycobiome, members of the Saccharomycetales and Mucorales were the most common genera, in contrast to the outdoor mycobiome, which was mainly dominated by Pucciniales, Capnodiales, Agaricales, and Chaetothyriales (Fig. 3a). The true yeasts of the Saccharomycetales were considerably more abundant in the indoor environments. Malasseziales, a basidiomycete yeast order, were also somewhat more abundant indoors (Fig. 3a). We annotated the 1,253 most abundant OTUs (OTUs with >20 sequences) into different growth and life forms, which revealed that yeasts, dimorphic yeasts, and molds were considerably more abundant in indoor environments, while saprotrophs, plant pathogens, and lichens dominated relatively more in the outdoor samples (Fig. 3b).

**FIG 4:**
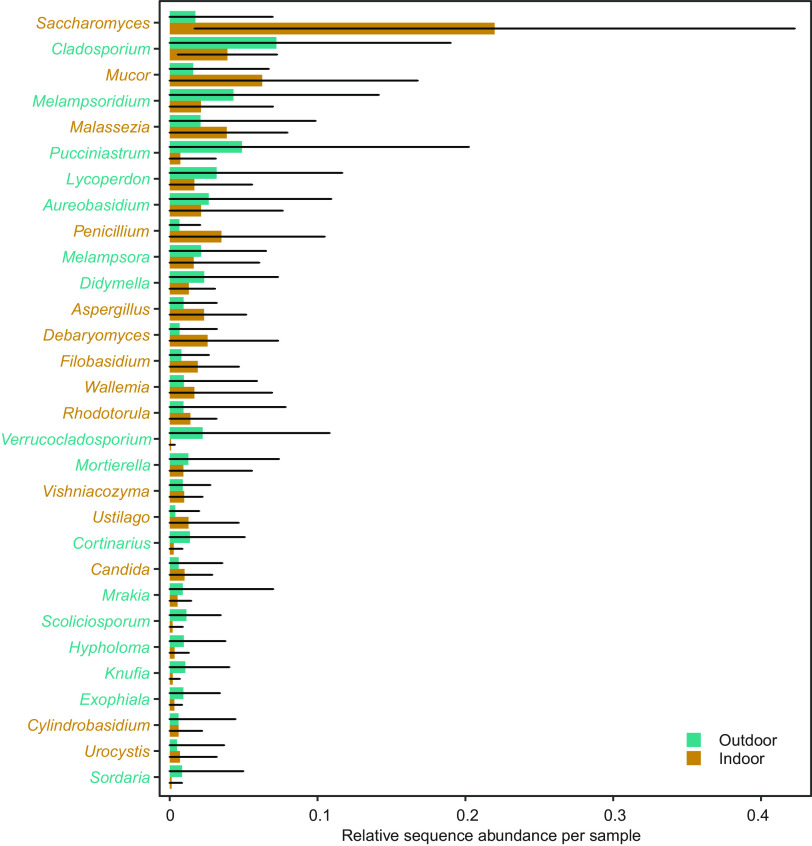
The 30 most abundant genera in the data set, with their average sequence abundances across indoor and outdoor samples in the 125 daycare centers displayed. For the indoor samples, a mean value from the merged bathroom and main room sample was used for the calculations. Genera with higher indoor abundances are displayed in brown, while genera with higher outdoor abundances are shown in cyan. The black lines indicate standard errors.

Among the top 30 genera detected in this study, measured in sequence abundance in a balanced indoor/outdoor data set (where the sequence abundances of the two indoor samples were averaged), many had a clear affinity for either indoor or outdoor environments (Fig. 4). Ten genera, namely, Aspergillus, *Candida*, *Debaryomyces*, *Filobasidium*, *Malassezia*, *Mortierella*, *Mucor*, *Penicillium*, *Rhodotorula*, *Saccharomyces*, and *Wallemia*, had a clear affinity for indoor environments. *Saccharomyces* was by far the most abundant genus in the indoor environment, with an ∼12.5-times-higher abundance indoors than outdoors. In contrast, plant pathogens like *Melampsora*, *Puccinastrum*, and *Melampsoridium* were relatively more common in the outdoor samples. Interestingly, some genera with an affinity for the outdoor environment, like *Verrucocladosporium*, *Scoliciosporum*, and *Sordaria*, were almost exclusively present in the outdoor samples, while others, like *Cladosporium*, *Melampsoridium*, and *Lycoperdon*, were also abundant in the indoor environment.

The 30 most abundant OTUs in the indoor environment are presented in Table 4. An OTU annotated as Saccharomyces paradoxus (98.4% identity to the reference sequence under GenBank accession no. AJ229059) was by far the most abundant OTU, being present in 98.1% of the indoor samples and making up 22.3% of all sequences. The three next most abundant OTUs were molds, with high identity to reference sequences of Mucor plumbeus, (98.3%), Cladosporium delicatulum, (100%), and Penicillium aethiopicum (100%). Among the 30 most abundant OTUs in the indoor samples, 12 were annotated as yeasts in the orders Saccharomycetales, Filobasidiales, Malasseziales, Sporidiobolales, Tremellales, and Kriegeriales. Also, several plant pathogens appeared among the most abundant indoor fungi, likely originating from outdoor sources (Table 4).

**TABLE 4:** Overview of the 30 most abundant indoor OTUs, as revealed by the sequence proportions of all indoor sequences in the rarefied data set (566,324)^*a*^

Species	GenBank accession no.	% identity	Order	Nutritional mode	Sequence proportion (%)	% occurrence
Saccharomyces paradoxus	AJ229059	98.4	Saccharomycetales	Yeast	22.26	98.1
*Mucor plumbeus*	JF723573	98.3	Mucorales	Mold	4.98	75.8
*Cladosporium delicatulum*	HM148081	100	Capnodiales	Mold	3.85	99.1
*Penicillium aethiopicum*	AY371635	100	Eurotiales	Mold	2.88	91.9
*Naganisha* sp. 1^*b*^	MT303157	100	Filobasidiales	Yeast	2.74	87.0
Debaryomyces hansenii	AB053101	99	Saccharomycetales	Yeast	2.59	90.0
*Malassezia restricta*	AY743636	98.9	Malasseziales	Yeast	2.31	94.8
*Melampsoridium betulinum*	AF125177	100	Pucciniales	Plant pathogen	2.15	59.5
*Naganisha* sp. 2^*b*^	MN796318	100	Filobasidiales	Yeast	1.85	81.3
*Aureobasidium pullulans*	AJ244232	100	Dothideales	Mold/plant biotroph	1.79	85.3
Aspergillus *appendiculatus*	HE615132	100	Eurotiales	Mold	1.61	85.8
*Filobasidium magnum*	AF190008	100	Filobasidiales	Yeast	1.49	82.9
*Melampsora epitea*	JF825968	98.7	Pucciniales	Plant pathogen	1.48	51.9
*Lycoperdon pyriforme*	DQ112557	100	Agaricales	Wood/soil saprotroph	1.32	54.5
Rhodotorula mucilaginosa	AF444541	99.5	Sporidiobolales	Yeast	1.27	83.2
*Didymella gardeniae*	FJ427003	97.8	Pleosporales	Plant pathogen	1.19	82.5
*Ustilago nunavutica*	KF381025	100	Ustilaginales	Plant pathogen	1.01	48.3
*Vishniacozyma victoriae*	AF444469	100	Tremellales	Yeast	0.80	89.3
Cryptococcus sp.	KP714547	100	Tremellales	Yeast	0.76	69.0
*Urocystis agropyri*	KX057781	98.9	Urocystidales	Plant pathogen	0.69	37.4
*Pucciniastrum areolatum*	DQ445901	100	Pucciniales	Plant pathogen	0.67	33.9
*Cylindrobasidium evolvens*	UDB016342	100	Agaricales	Wood saprotroph	0.61	53.8
*Wallemia muriae*	AY302534	98.6	Wallemiales	Mold	0.59	57.1
*Bovista plumbea*	DQ112613	100	Agaricales	Soil saprotroph	0.55	32.5
*Kendrickiella phycomyces*	KJ407018	98.4	Eurotiales	Possible saprotroph	0.54	69.9
Xylariales sp.	JQ070614	100	Xylariales	Saprotroph	0.52	57.3
*Malassezia globosa*	KP825429	99.3	Malasseziales	Yeast	0.50	79.9
Cryptococcus *uniguttulatus*	AF444302	100	Tremellales	Yeast	0.47	41.2
*Mucor abundans*	JN206111	97.5	Mucorales	Mold	0.46	10.2
Kriegeriaceae sp.	KF414531	97.4	Kriegeriales	Yeast	0.45	52.1

a The taxonomic affiliation of the best BLAST match is shown in the first column, along with the GenBank accession number and sequence identity. The last column provides proportional information about occurrences among the 422 indoor samples.

b One hundred percent sequence identity to numerous *Naganisha* species.

## DISCUSSION

### Factors influencing the indoor mycobiome.

We observed a clear separation between the outdoor and indoor mycobiomes across the 125 Norwegian daycare centers and that numerous variables associated with both the outdoor climate and the indoor environment together influenced the indoor mycobiome. We observed a similar pattern in a recent study of private homes across the same climatic gradients in Norway (13). Likewise, Barberán et al. reported a similar trend from analyses of house dust mycobiomes throughout the United States (24). However, other preceding studies pointed out that indoor air and dust mostly consist of outdoor fungi that have spread into buildings through the ventilation system, windows, or doors (12, 29, 30). For example, Shin et al. concluded that human activity had little influence on the indoor fungal community composition in daycare centers in Seoul, South Korea (30). Similarly, in a study investigating indoor fungi in a housing facility in California, Adams et al. showed that the outdoor air and not the residents structured the indoor mycobiome (12). Interestingly, in our recent study on the seasonality of the indoor mycobiome, the indoor environment was more influenced by the outdoor fungal diversity during summer and fall (10). Thus, as the community scientists in the present study performed sampling during early spring, we may have detected a stronger influence of indoor variables than, e.g., Shin et al. (30), where samples were collected from August to October in a comparable climate in South Korea.

According to the CCA, the number of children in daycares accounted for some of the overall variation in the indoor mycobiome composition, together with the year of construction and the occurrence of pests and rodents. Furthermore, the variables building type, number of departments, room (main room versus bathroom), and type of daycare correlated significantly with the mycobiome composition in single-factor analyses. Taken together, these results indicate that the organization of daycare centers and building features influence the indoor mycobiome composition. In addition to building variables, regional climate-related factors, such as the maximum temperature in June, the mean temperature of the coldest quarter, and total insolation, also correlated significantly with the indoor mycobiome composition. Longitude, an approximation for regional climate variability, also had explanatory power. Throughout most of Norway, longitude mirrors a climate gradient from oceanic and humid areas in the west to areas with drier, colder, and high-temperature seasonality conditions in the east. Our findings mirror the observations by Barberán et al. (24) and Martin-Sanchez et al. (13), where the regional climate was found to be important for the indoor mycobiome. The climate factors most likely have indirect effects on the indoor fungi, as they influence and structure the outdoor fungi that disperse into buildings.

Although several of the assessed variables were significantly related to the composition of the indoor mycobiome, only a small fraction of the variation in the indoor mycobiome composition was accounted for (7%). In theory, there may be additional factors that were not included in this study, such as the humidity level, heating systems, or other building factors, that might account for some of the unexplained variation. However, the low level of explanatory power is not a feature unique to this study but rather a common trend across most fungal diversity studies (13, 31). Fungal communities are largely assembled through colonization by spore dispersal, which, to a large extent, is a random process. Because of this, it is generally difficult to account for all variables structuring the fungal community composition.

### Taxonomic composition of daycare mycobiomes.

The most marked taxonomic difference in the indoor and outdoor dust mycobiomes was the predominance of yeasts and molds inside the daycare centers. *Saccharomyces* was by far the most abundant genus in our study and had a clear affinity for indoor environments. Based on the taxonomic annotation, what appears to be Saccharomyces paradoxus was clearly the most abundant fungal OTU in our data set. Compared to Saccharomyces cerevisiae, the mesophilic yeast S. paradoxus has a more northern distribution (32). In addition to its natural habitats, *Saccharomyces* may partly be derived from food but has also been found as one of the most abundant genera in the human gut (33) and on children’s skin (34). Other true yeasts, such as *Debaryomyces* and *Candida* (2 among the top 50), also had a clear affinity for indoor environments in the studied daycares. *Candida* and *Debaryomyces* are among the most widespread genera associated with external (skin) and internal (mouth and digestive tract) parts of the human body (35). It is well documented that *Candida* is particularly associated with children and can in some cases cause oral thrush (massive *Candida* growth in the mouth and throat) in the first years of life (36). The lipophilic basidiomycete yeast *Malassezia*, a widespread genus on human skin (18), and *Rhodotorula*, another basidiomycete yeast associated with the human body (35), were also prevalent in the studied daycares. *Malassezia* as well as *Candida* are known to be associated with inflammatory skin disorders such as seborrheic dermatitis and atopic dermatitis in childhood as well as adulthood (37, 38). However, *Malassezia* most often has a commensal role, as it is widespread on healthy skin. For instance, 11 of the 14 known *Malassezia* species were associated with different parts of the skin of 14 healthy adults (20), indicating that human skin is colonized with a wide range of *Malassezia* species. On children’s skin, a dominance of the species Malassezia globosa has been observed (18), which was among our most abundant OTUs according to the taxonomic annotation (99.3% match to the reference sequence). We hypothesize that the yeasts dominating the indoor daycare mycobiomes are mainly derived from different parts of the human body. The high density of children and close physical contact may lead to the easy and fast transmission of yeasts in daycares, possibly explaining the increased concentrations of these species indoors.

In addition to the indoor enrichment of yeasts, several extremotolerant molds, such as *Mucor*, *Penicillium*, Aspergillus, and *Wallemia*, were also abundant in the indoor environment. These genera are widespread and prevalent in most indoor environments (13, 24, 39) and grow rapidly on organic materials. All of these taxa are sometimes detected on and in the human body as well (18). *Cladosporium*, another abundant mold in indoor environments, was prevalent in both indoor and outdoor samples and might largely be dispersed from outdoor sources. Although no direct cause-effect relationship has been established, some of these mold taxa were abundant in houses with children with allergies and respiratory diseases (15, 40). However, precautions have to be taken when interpreting the DNA metabarcoding data quantitatively. Our DNA metabarcoding analysis does not inform about absolute quantities but provides proportional data, which may lead to spurious patterns. For example, if relatively small amounts of fungal DNA occur in the indoor compared to the outdoor samples, outdoor taxa that are able to spread indoors will occur with high relative abundances here and appear as fungi with a preference for indoor conditions. In line with this, several obviously outdoor OTUs, like Melampsoridium betulinum (100% BLAST match), Melampsora epitea (98.7%), and Ustilago nunavutica (100%), were among the most prevalent indoor fungi. Quantitative analyses, such as quantitative PCR (qPCR), will be helpful to better separate and identify the sources of the indoor fungi.

### Concluding remarks.

Taken together, we conclude that the indoor mycobiomes of Norwegian daycare centers are dominated by yeasts and molds and that a multitude of factors structure their composition. For the current study, dust samples were obtained during a relatively short time window during the spring of 2018. From other studies, we know that there is extensive temporal variability (10), which is not accounted for here. Moreover, sampling at approximately the same time throughout Norway, a country that spans a wide range of latitudes and longitudes, implies that the outdoor climate, vegetation, and fungal communities are in different (phenological) growth phases. Such differences can influence which fungi we recovered from the different regions that we sampled. Indeed, the variable (sampling) month was significantly correlated with the fungal community composition, but it accounted for only a small amount of the variation. Most likely, indoor fungi dominated by yeasts and molds can be sampled in higher proportions during winter and spring in the Norwegian climate, while the influence of outdoor fungi on the indoor mycobiome will be greater during the growth and sporulation periods of most mushrooms (summer and fall). Hence, a sampling time during the winter period may be even more representative of the specific indoor fungal community in future studies. We also note that our dust samples may include both dormant (live) and dead fungal spores as well as dead and living hyphal fragments. By using our DNA metabarcoding approach, we cannot differentiate between living and dead fungal material that might have accumulated over longer time periods.

In the current study, we carried out a community science sampling approach for obtaining material from daycare centers. In line with our previous study (13), only a few outlier samples occurred, and the indoor and outdoor dust samples were largely separated in species composition, indicating a small influence of sampling bias. Moreover, very few samples were discarded due to low DNA yields. Our results supported that community science sampling is a powerful approach to obtain samples from a wide geographic area during a short time span. We advocate for further community science studies for evaluating biological and chemical air pollutants, which will also help to raise public awareness of air quality problems in buildings.

## MATERIALS AND METHODS

### Sampling.

A list of Norwegian daycare centers was retrieved from the Norwegian Directorate of Health (Helsedirektoratet). The list was sorted alphabetically after counties and municipalities, and the first five municipalities in each county were selected for the study. Likewise, the first 3 to 4 daycare centers in each of these municipalities were chosen as candidate sites for dust sampling. Sampling kits containing five FLOQSwabs (Copan Italia SPA, Brescia, Italy) and a questionnaire were sent to the selected daycare centers with specifications to perform dust sampling on doorframes: (i) outdoor, (ii) main room, and (iii) bathroom. If the daycare had two different departments, we requested that sampling be repeated in (iv) the main room and (v) the bathroom of the second department as well. Overall, 572 samples were retrieved from a total of 125 studied daycare centers (Fig. 1), and upon arrival, the swabs were stored at −80°C until DNA extraction.

### DNA extraction and metabarcoding.

Samples were prepared and DNA was extracted using the EZNA soil DNA kit (Omega Bio-Tek, Norcross, GA, USA). The tips of the swabs were placed into disruptor tubes by using a sterilized scissor. The empty swab tubes were filled with 800 μL SLX-Mlus buffer to collect the remaining dust before being transferred to the disruptor tubes. The samples were homogenized twice for 1 min at 30 Hz using a TissueLyser (Qiagen, Hilden, Germany) and stored at −20°C until further processing.

DNA extraction and metabarcoding library preparation were performed according to methods described previously by Estensmo et al. (10). Briefly, samples were thawed at 70°C, followed by incubation for 10 min at the same temperature, and homogenized twice for 1 min at 30 Hz using the TissueLyser. The samples were then cooled on ice before adding 600 μL chloroform, vortexed, and centrifuged at 13,000 rpm for 5 min at room temperature (RT). The aqueous phase was transferred to a new 1.5-mL tube, and an equal volume of XP1 buffer was added before vortexing. The extract was transferred to a HiBind DNA minicolumn and further processed according to the manufacturer’s guidelines. The DNA was eluted in 50 μL elution buffer.

We targeted the ITS2 region with the forward primer ITS4 (5′-xCTCCGCTTATTGATATG) (41) and the modified reverse primer gITS7 (5′-xGTGARTCATCGARTCTTTG) (42) (with barcodes x ranging from 6 to 9 bp). The amplification mix contained 2 μL DNA template, 14.6 μL MilliQ water, 2.5 μL 10× Gold buffer, 0.2 μL deoxynucleoside triphosphates (dNTPs) (25 nM), 1.5 μL reverse and forward primers (10 μM), 2.5 μL MgCl_2_ (50 mM), 1.0 μL bovine serum albumin (BSA) (20 mg/mL), and 0.2 μL AmpliTaq Gold polymerase (5 U/μL). DNA was amplified by an initial denaturation step at 95°C for 5 min, followed by 32 cycles of denaturation at 95°C for 30 s, annealing at 55°C for 30 s, and elongation at 72°C for 1 min. A final elongation step was included at 72°C for 10 min. Amplicons were normalized using the SequalPrep normalization plate kit (Invitrogen, Thermo Fisher Scientific, Waltham, MA, USA) and eluted in 20 μL elution buffer. The resulting PCR products were processed into seven libraries of 96 samples using a combination of 96 tagged primers. Each library included 10 technical replicates (DNA extracts from the same 10 dust samples amplified and sequenced independently for each library), one mock community (artificial fungal community composed of DNA in 1-ng/μL equimolar concentrations from Mycena belliarum, Pycnoporellus fulgens, Serpula similis, and Pseudoinonotus dryadeus), negative DNA controls (using a clean swab as the starting material), and negative PCR controls. The 96 PCR products within each library were pooled, concentrated, and purified using Agencourt AMPure XP magnetic beads (Beckman Coulter, CA, USA). The quality of the purified pools was measured using Qubit (Invitrogen, Thermo Fisher Scientific, Waltham, MA, USA). The seven libraries were barcoded with Illumina adapters, spiked with PhiX, and sequenced in three Illumina (San Diego, CA, USA) MiSeq lanes with 2-by-250-bp paired-end reads at Fasteris SA (Plan-les-Ouates, Switzerland).

### Bioinformatics.

The bioinformatics analyses were performed according to methods described previously by Estensmo et al. (10). Briefly, raw sequences were demultiplexed independently using CUTADPT (43) allowing no mismatches between barcode tags and the sequencing primer, and sequences of <100 bp were discarded. DADA2 (44) was used to filter low-quality reads and for error correction. We then merged the error-corrected sequences using a minimum overlap of 5 bp. Chimeras were removed using the bimera algorithm, using default parameters, implemented in DADA2. The resulting amplicon sequence variant (ASV) table was further clustered into 10,955 operational taxonomic units (OTUs) using VSEARCH (45) at 97% similarity. LULU (46) was used with default settings to correct for potential OTU oversplitting. Taxonomy was assigned using BLAST (47) and to the final OTU table using the UNITE database (48). Sequences with no match to any known fungal sequences and samples with fewer than 10 OTUs were discarded from downstream analyses. The final raw data set (without technical replicates and controls) contained 7,399 OTUs and 22,655,516 reads from 572 samples. The number of reads per sample varied from 19 to 182,266, with a mean value of 39,608. The number of OTUs per sample varied from 10 to 863, with a mean value of 257.

### Environmental variables.

Metadata about building features, type of daycare (nature, farm, combined, and departments), and occupancy of each daycare were provided by the volunteers in a questionnaire that was delivered together with the samples (Table 1). The location of daycare centers and complete addresses were provided, and the corresponding geographic coordinates (latitude and longitude) were retrieved. Based on these coordinates, relevant environmental variables were extracted and kindly provided by the authors of a recent study modeling the vegetation types in Norway (28). From this extensive set of environmental variables (>30), a subset of noncolinear variables (|*r*| > 0.6) was selected for further analyses (Table 1).

### Annotation of fungal (OTU) growth characteristics.

We annotated the 1,593 most abundant OTUs, defined as those with >20 sequences and taxonomic annotation at the species, genus, or family level, into growth forms/nutritional modes based on literature surveys and information available in the UNITE database (48). Species/genera/families having unknown, dubious, or multiple growth forms/nutritional modes were not included. A complete list of our annotations can be found in Table S1 in the supplemental material.

### Statistics.

Statistical analyses were performed in R (49). Given that DNA metabarcoding analyses of samples with low DNA yields may introduce biases during the wet-lab analyses and sequencing, we controlled the consistency of our results. The above-mentioned mock community was included to check for tag switching and species recovery and as controls for library preparation, sequencing, and bioinformatics analyses. The similarity of the technical replicates was evaluated by nonmetric multidimensional scaling (NMDS) using the metaMDS function from the VEGAN package version 2.4-2 (50), and the results were visualized using GGPLOT2 (51) (Fig. S1). As visualized in Fig. S1, the distances between biological replicates are generally markedly higher than those between technical replicates. Next, all the samples in the complete data set were rarefied to 1,342 sequences using the function rrarefy (VEGAN). Fourteen samples were discarded for downstream statistical analyses due to a lower sequencing depth.

To visualize and investigate patterns in OTU composition in relation to environmental variables, we performed global nonmetric multidimensional scaling (GNMDS) using the VEGAN package and the settings recommended by the authors. To ensure the reliability of the results, a detrended correspondence analysis (DCA) was performed in parallel. Extreme outliers common to both ordinations were manually inspected and subsequently removed from the data set before the analyses were repeated. Both ordination analyses revealed the same overall pattern (data not shown), and here, we focus on the GNMDS analyses. The GNMDS was scaled into half-change units and subjected to varimax rotation using principal-component analyses (PCA). To confirm convergence, the two best solutions of the GNMDS were compared using Procrustes comparisons with 999 permutations (correlation, 0.99; *P* = 0.001). The ordinations were first conducted on the entire data set containing both indoor and outdoor samples, where a clear pattern was observed. Thereafter, a data set containing only indoor samples from main rooms and bathrooms was extracted, and the ordinations were conducted on this data set using the same settings and correlation as those for the Procrustes comparisons. The following analyses were conducted on the indoor data set only. The envfit function in VEGAN (i.e., the fit [*R*^2^] of each variable assessed by a Monte-Carlo analysis with 999 permutations) was used to fit the environmental variables building type, average construction year, June temperature, longitude, mean temperature of the coldest quarter, month, numbers of departments and children, the presence of rodents (pests), proximity to all types of water, proximity to the coast, room type, and type of daycare to the GNMDS. The numerical variables were visualized using the vectors from the output from the envfit function. We further performed variation partitioning by CCA (canonical correspondence analysis) with 999 permutations to quantify the components of variation by the variables mentioned above, with forward selection, as implemented in VEGAN.

To investigate OTU richness trends, a linear mixed-effect model was applied using the NLME package (52), including daycare identifier as a random contribution. Colinear variables were excluded as described above (|*r*| > 0.6); however, to further avoid multicolinearity in the mixed-effect model, the corvif function described previously by Zuur et al. was applied, using a threshold of 2.5 (53). Backward stepwise model selection was performed based on the Akaike information criterion (AIC). The distributions of the 30 most abundant genera across indoor and outdoor samples were visualized. In addition, bar charts were made based on relative abundances from the rarified OTU table and fungal annotations, comparing the indoor and outdoor environments. To obtain a balanced indoor/outdoor data set, one indoor “observation” and one outdoor observation, we collapsed the indoor samples to one observation by using the average values from the indoor samples (main room and bathroom).

### Data availability.

Our initial data set as well as the final rarefied data set are available at Dryad (https://doi.org/10.5061/dryad.sn02v6x5s) together with information about metadata, scripts for bioinformatics and statistics, taxonomic annotations, and growth form/nutritional mode annotations.
