# Supplementary material for: The Indoor Mycobiomes of Daycare Centers Are Affected by Occupancy and Climate
Source: Appl Environ Microbiol. 2022 Mar 22;88(6):e02113-21. doi: 10.1128/aem.02113-21 (PMC8939353; doi:10.1128/aem.02113-21)
Supplement: Supplemental file 1 — Fig. S1 and description of Table S1. Download aem.02113-21-s0001.pdf, PDF file, 0.1 MB [file aem.02113-21-s0001.pdf]

Supplementary material

Supplementary tables

Supplementary table 1. The complete list of our annotations of fungal (OTUs) growth characteristics and their frequency (the percentage of samples they are present in) based on the rarified OTU table.

See excel table "Supplementary table 1".

Supplementary figures

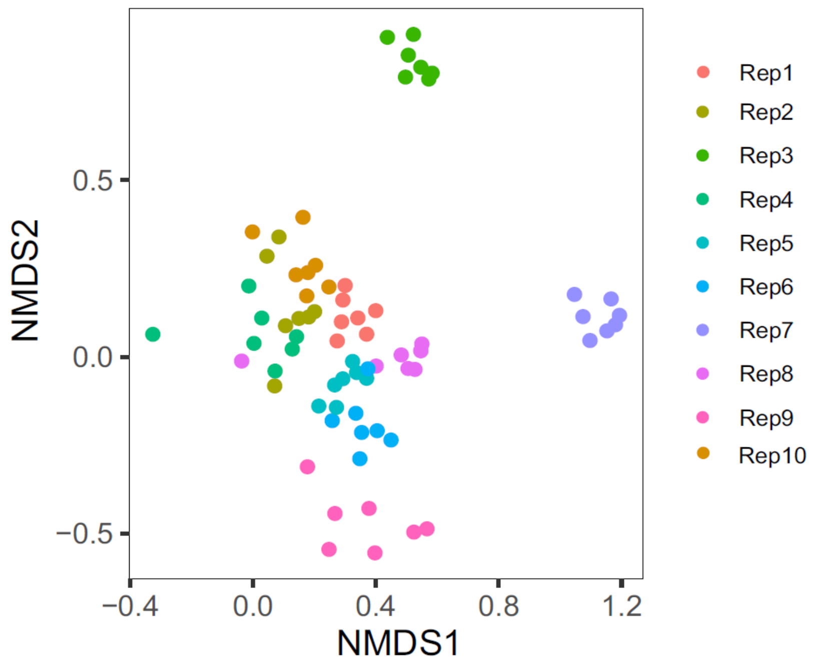

S1. Nonmetric multidimensional scaling (NMDS) ordination plot of technical replicates. Each point represents one sample from one library, and the color separates the different replicates (DNA extract from 10 different dust samples, one of each included in each sequencing library). The plot illustrates that the technical replicates (same color) cluster together (with a few exceptions) and that the distances between biological replicates (different colors) are generally higher than between technical replicates.
